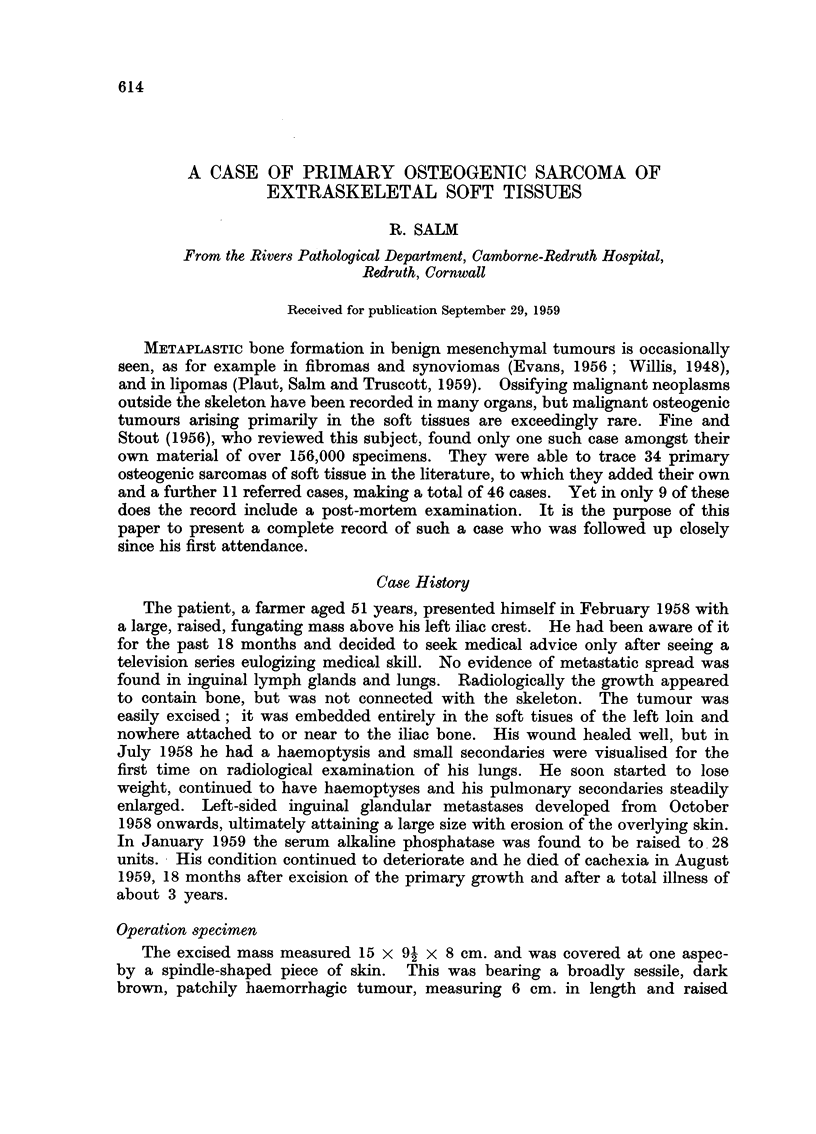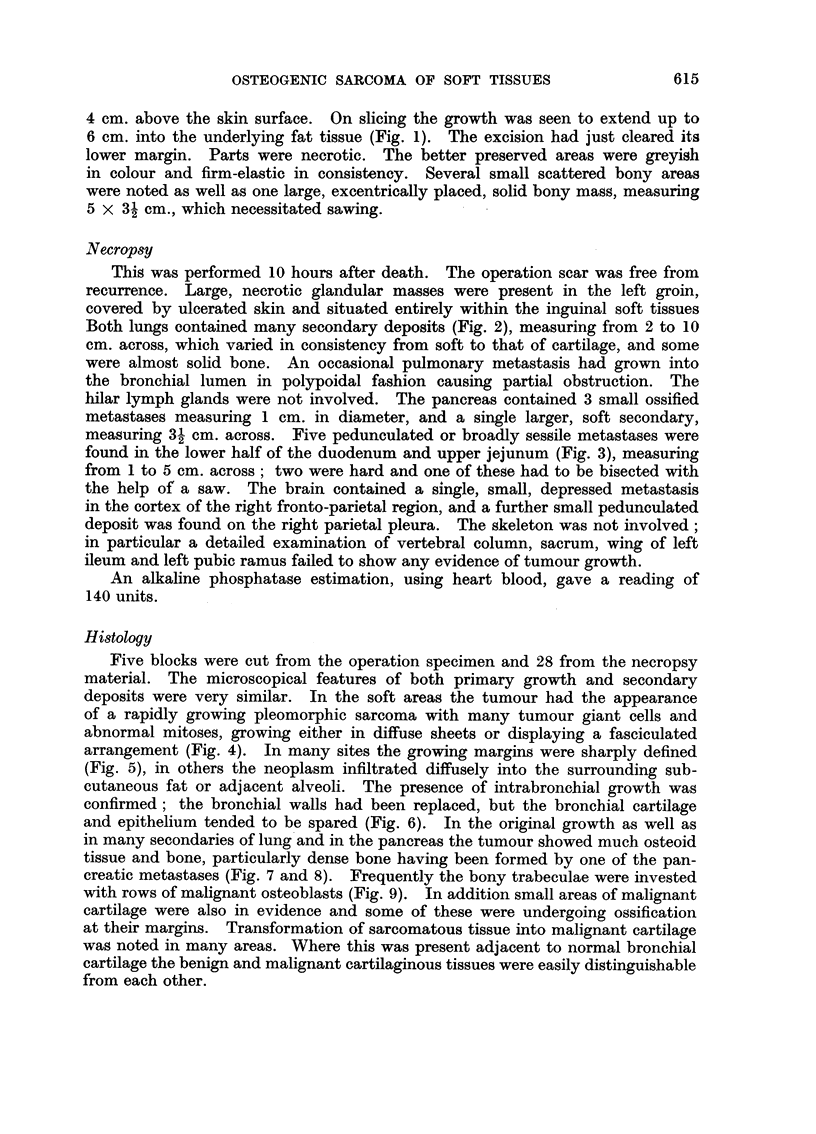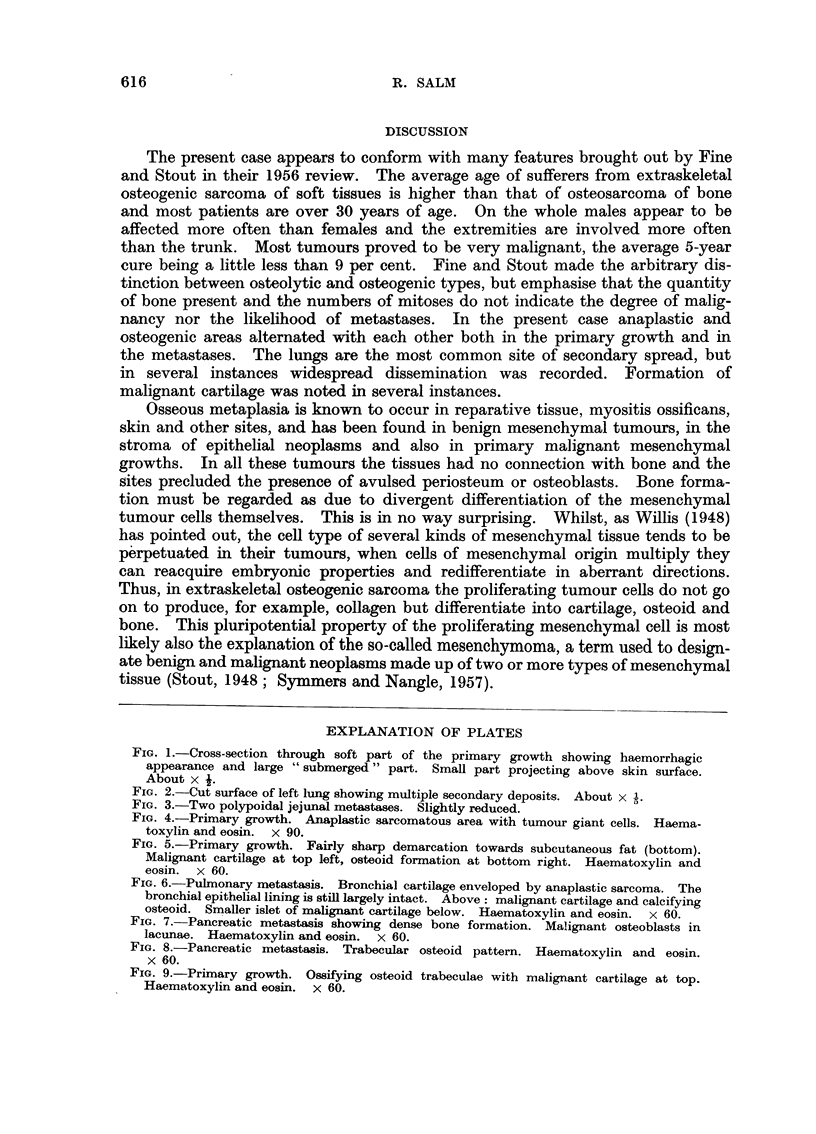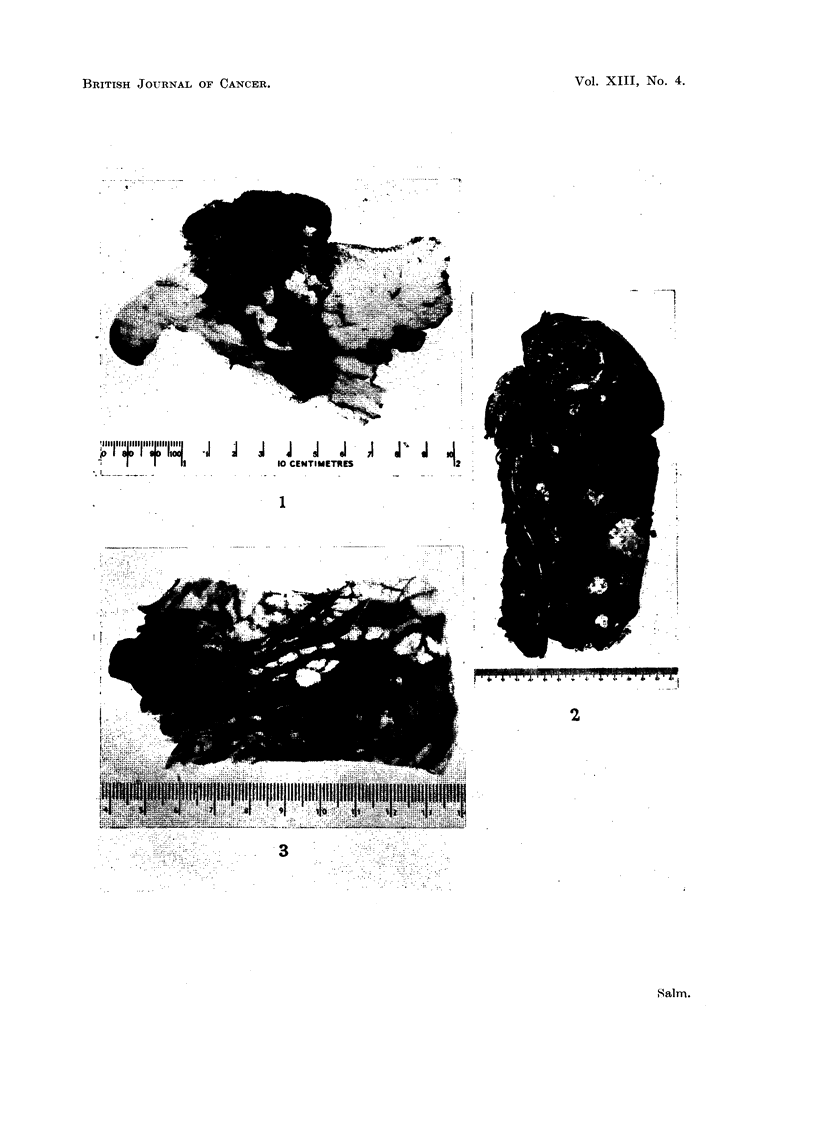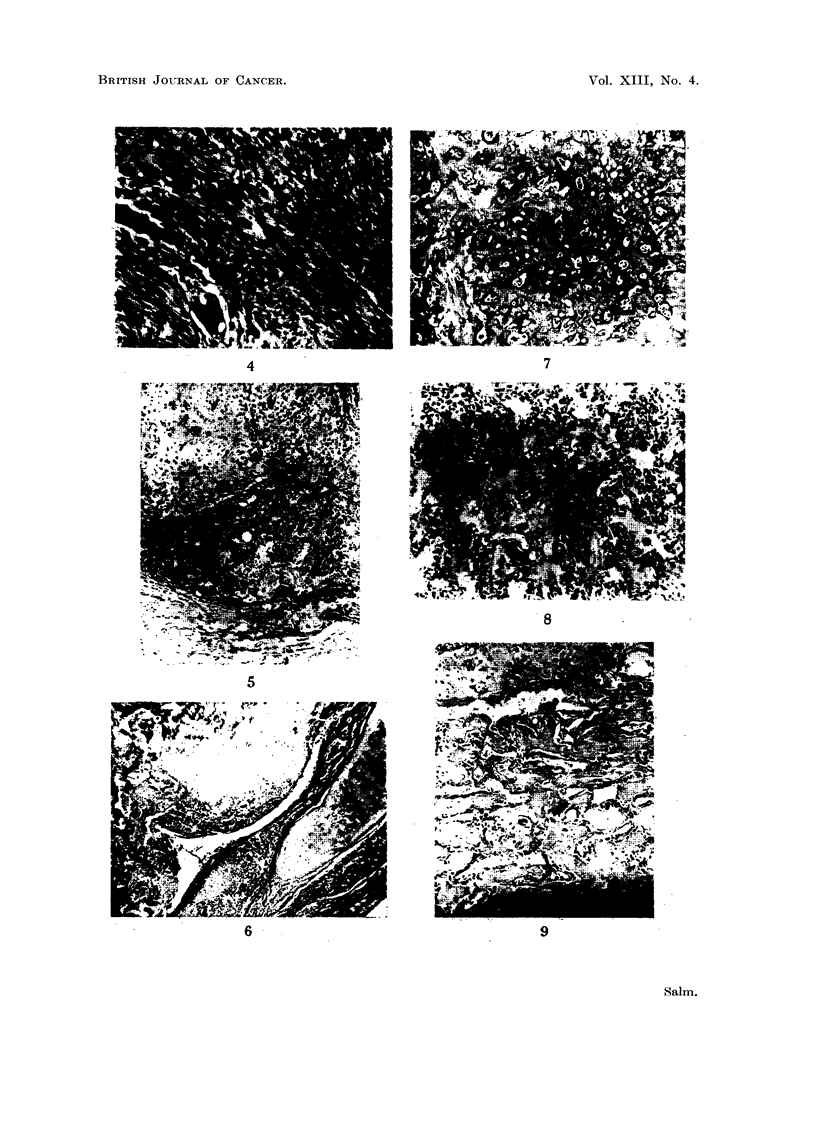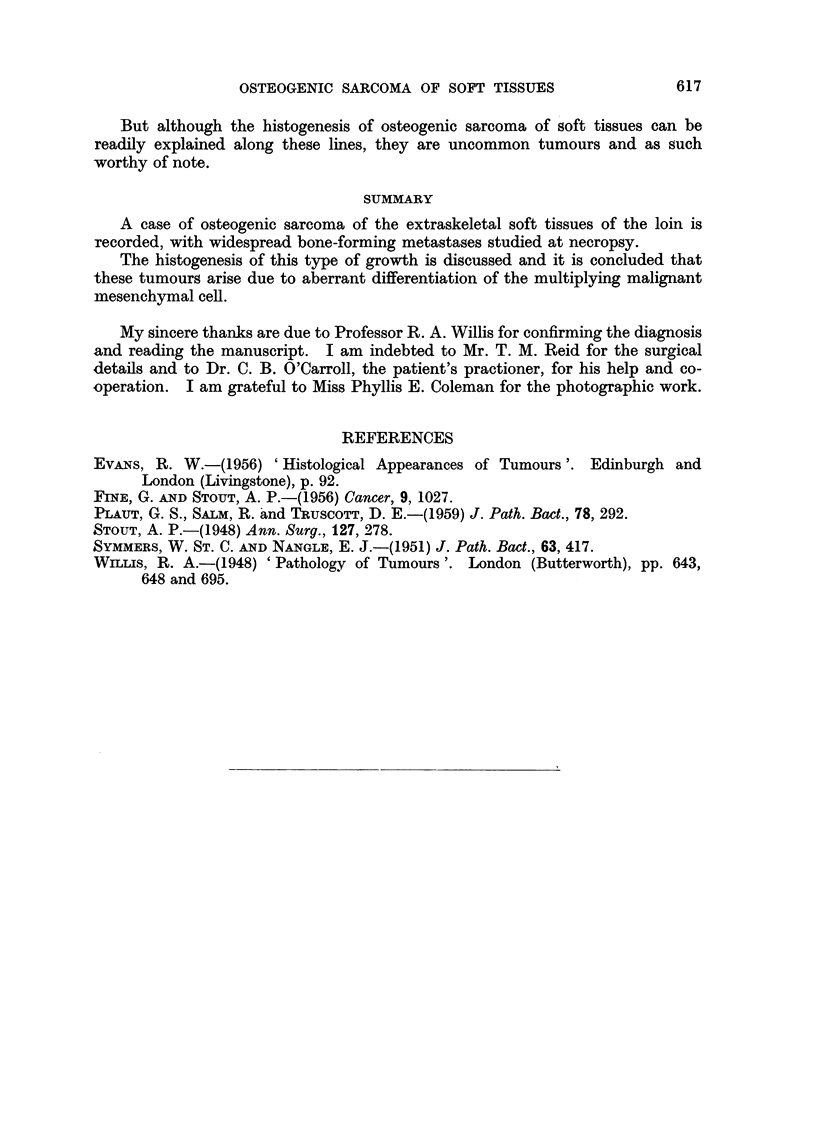# A Case of Primary Osteogenic Sarcoma of Extraskeletal Soft Tissues

**DOI:** 10.1038/bjc.1959.66

**Published:** 1959-12

**Authors:** R. Salm

## Abstract

**Images:**


### A CASE OF PRIMARY OSTEOGENIC SARCOMA OF EXTRASKELETAL SOFT TISSUES

R. SALM

*From the Rivers Pathological Department, Camborne-Redruth Hospital, Redruth, Cornwall*

Received for publication September 29, 1959

METAPLASTIC bone formation in benign mesenchymal tumours is occasionally seen, as for example in fibromas and synoviomas (Evans, 1956; Willis, 1948), and in lipomas (Plaut, Salm and Truscott, 1959). Ossifying malignant neoplasms outside the skeleton have been recorded in many organs, but malignant osteogenic tumours arising primarily in the soft tissues are exceedingly rare. Fine and Stout (1956), who reviewed this subject, found only one such case amongst their own material of over 156,000 specimens. They were able to trace 34 primary osteogenic sarcomas of soft tissue in the literature, to which they added their own and a further 11 referred cases, making a total of 46 cases. Yet in only 9 of these does the record include a post-mortem examination. It is the purpose of this paper to present a complete record of such a case who was followed up closely since his first attendance.

### *Case History*

The patient, a farmer aged 51 years, presented himself in February 1958 with a large, raised, fungating mass above his left iliac crest. He had been aware of it for the past 18 months and decided to seek medical advice only after seeing a television series eulogizing medical skill. No evidence of metastatic spread was found in inguinal lymph glands and lungs. Radiologically the growth appeared to contain bone, but was not connected with the skeleton. The tumour was easily excised; it was embedded entirely in the soft tisues of the left loin and nowhere attached to or near to the iliac bone. His wound healed well, but in July 1958 he had a haemoptysis and small secondaries were visualised for the first time on radiological examination of his lungs. He soon started to lose weight, continued to have haemoptyses and his pulmonary secondaries steadily enlarged. Left-sided inguinal glandular metastases developed from October 1958 onwards, ultimately attaining a large size with erosion of the overlying skin. In January 1959 the serum alkaline phosphatase was found to be raised to 28 units. His condition continued to deteriorate and he died of cachexia in August 1959, 18 months after excision of the primary growth and after a total illness of about 3 years.

### *Operation specimen*

The excised mass measured $15 \times 9\frac{1}{2} \times 8$ cm. and was covered at one aspec- by a spindle-shaped piece of skin. This was bearing a broadly sessile, dark brown, patchily haemorrhagic tumour, measuring 6 cm. in length and raised

4 cm. above the skin surface. On slicing the growth was seen to extend up to 6 cm. into the underlying fat tissue (Fig. 1). The excision had just cleared its lower margin. Parts were necrotic. The better preserved areas were greyish in colour and firm-elastic in consistency. Several small scattered bony areas were noted as well as one large, excentrically placed, solid bony mass, measuring $5 \times 3\frac{1}{2}$ cm., which necessitated sawing.

*Necropsy*

This was performed 10 hours after death. The operation scar was free from recurrence. Large, necrotic glandular masses were present in the left groin, covered by ulcerated skin and situated entirely within the inguinal soft tissues Both lungs contained many secondary deposits (Fig. 2), measuring from 2 to 10 cm. across, which varied in consistency from soft to that of cartilage, and some were almost solid bone. An occasional pulmonary metastasis had grown into the bronchial lumen in polypoidal fashion causing partial obstruction. The hilar lymph glands were not involved. The pancreas contained 3 small ossified metastases measuring 1 cm. in diameter, and a single larger, soft secondary, measuring $3\frac{1}{2}$ cm. across. Five pedunculated or broadly sessile metastases were found in the lower half of the duodenum and upper jejunum (Fig. 3), measuring from 1 to 5 cm. across ; two were hard and one of these had to be bisected with the help of a saw. The brain contained a single, small, depressed metastasis in the cortex of the right fronto-parietal region, and a further small pedunculated deposit was found on the right parietal pleura. The skeleton was not involved ; in particular a detailed examination of vertebral column, sacrum, wing of left ileum and left pubic ramus failed to show any evidence of tumour growth.

An alkaline phosphatase estimation, using heart blood, gave a reading of 140 units.

*Histology*

Five blocks were cut from the operation specimen and 28 from the necropsy material. The microscopical features of both primary growth and secondary deposits were very similar. In the soft areas the tumour had the appearance of a rapidly growing pleomorphic sarcoma with many tumour giant cells and abnormal mitoses, growing either in diffuse sheets or displaying a fasciculated arrangement (Fig. 4). In many sites the growing margins were sharply defined (Fig. 5), in others the neoplasm infiltrated diffusely into the surrounding subcutaneous fat or adjacent alveoli. The presence of intrabronchial growth was confirmed ; the bronchial walls had been replaced, but the bronchial cartilage and epithelium tended to be spared (Fig. 6). In the original growth as well as in many secondaries of lung and in the pancreas the tumour showed much osteoid tissue and bone, particularly dense bone having been formed by one of the pancreatic metastases (Fig. 7 and 8). Frequently the bony trabeculae were invested with rows of malignant osteoblasts (Fig. 9). In addition small areas of malignant cartilage were also in evidence and some of these were undergoing ossification at their margins. Transformation of sarcomatous tissue into malignant cartilage was noted in many areas. Where this was present adjacent to normal bronchial cartilage the benign and malignant cartilaginous tissues were easily distinguishable from each other.

### DISCUSSION

The present case appears to conform with many features brought out by Fine and Stout in their 1956 review. The average age of sufferers from extraskeletal osteogenic sarcoma of soft tissues is higher than that of osteosarcoma of bone and most patients are over 30 years of age. On the whole males appear to be affected more often than females and the extremities are involved more often than the trunk. Most tumours proved to be very malignant, the average 5-year cure being a little less than 9 per cent. Fine and Stout made the arbitrary distinction between osteolytic and osteogenic types, but emphasise that the quantity of bone present and the numbers of mitoses do not indicate the degree of malignancy nor the likelihood of metastases. In the present case anaplastic and osteogenic areas alternated with each other both in the primary growth and in the metastases. The lungs are the most common site of secondary spread, but in several instances widespread dissemination was recorded. Formation of malignant cartilage was noted in several instances.

Osseous metaplasia is known to occur in reparative tissue, myositis ossificans, skin and other sites, and has been found in benign mesenchymal tumours, in the stroma of epithelial neoplasms and also in primary malignant mesenchymal growths. In all these tumours the tissues had no connection with bone and the sites precluded the presence of avulsed periosteum or osteoblasts. Bone formation must be regarded as due to divergent differentiation of the mesenchymal tumour cells themselves. This is in no way surprising. Whilst, as Willis (1948) has pointed out, the cell type of several kinds of mesenchymal tissue tends to be perpetuated in their tumours, when cells of mesenchymal origin multiply they can reacquire embryonic properties and redifferentiate in aberrant directions. Thus, in extraskeletal osteogenic sarcoma the proliferating tumour cells do not go on to produce, for example, collagen but differentiate into cartilage, osteoid and bone. This pluripotential property of the proliferating mesenchymal cell is most likely also the explanation of the so-called mesenchymoma, a term used to designate benign and malignant neoplasms made up of two or more types of mesenchymal tissue (Stout, 1948 ; Symmers and Nangle, 1957).

---

### EXPLANATION OF PLATES

FIG. 1.—Cross-section through soft part of the primary growth showing haemorrhagic appearance and large "submerged" part. Small part projecting above skin surface. About × $\frac{1}{2}$.

FIG. 2.—Cut surface of left lung showing multiple secondary deposits. About × $\frac{1}{5}$.

FIG. 3.—Two polypoidal jejunal metastases. Slightly reduced.

FIG. 4.—Primary growth. Anaplastic sarcomatous area with tumour giant cells. Haematoxylin and eosin. × 90.

FIG. 5.—Primary growth. Fairly sharp demarcation towards subcutaneous fat (bottom). Malignant cartilage at top left, osteoid formation at bottom right. Haematoxylin and eosin. × 60.

FIG. 6.—Pulmonary metastasis. Bronchial cartilage enveloped by anaplastic sarcoma. The bronchial epithelial lining is still largely intact. Above : malignant cartilage and calcifying osteoid. Smaller islet of malignant cartilage below. Haematoxylin and eosin. × 60.

FIG. 7.—Pancreatic metastasis showing dense bone formation. Malignant osteoblasts in lacunae. Haematoxylin and eosin. × 60.

FIG. 8.—Pancreatic metastasis. Trabecular osteoid pattern. Haematoxylin and eosin. × 60.

FIG. 9.—Primary growth. Ossifying osteoid trabeculae with malignant cartilage at top. Haematoxylin and eosin. × 60.

1

2

3

Salm.

4

7

5

8

6

9

Salm.

But although the histogenesis of osteogenic sarcoma of soft tissues can be readily explained along these lines, they are uncommon tumours and as such worthy of note.

SUMMARY

A case of osteogenic sarcoma of the extraskeletal soft tissues of the loin is recorded, with widespread bone-forming metastases studied at necropsy.

The histogenesis of this type of growth is discussed and it is concluded that these tumours arise due to aberrant differentiation of the multiplying malignant mesenchymal cell.

My sincere thanks are due to Professor R. A. Willis for confirming the diagnosis and reading the manuscript. I am indebted to Mr. T. M. Reid for the surgical details and to Dr. C. B. O'Carroll, the patient's practioner, for his help and co-operation. I am grateful to Miss Phyllis E. Coleman for the photographic work.

### REFERENCES

Evans, R. W.—(1956) 'Histological Appearances of Tumours'. Edinburgh and London (Livingstone), p. 92.
Fine, G. and Stout, A. P.—(1956) *Cancer*, **9**, 1027.
Plaut, G. S., Salm, R. and Truscott, D. E.—(1959) *J. Path. Bact.*, **78**, 292.
Stout, A. P.—(1948) *Ann. Surg.*, **127**, 278.
Symmers, W. St. C. and Nangle, E. J.—(1951) *J. Path. Bact.*, **63**, 417.
Willis, R. A.—(1948) 'Pathology of Tumours'. London (Butterworth), pp. 643, 648 and 695.